# Dynamic tissue perfusion assessment reflects associations between antihypertensive treatment and renal cortical perfusion in patients with chronic kidney disease and hypertension

**DOI:** 10.1007/s11255-018-1798-9

**Published:** 2018-01-27

**Authors:** Arkadiusz Lubas, Grzegorz Kade, Marek Saracyn, Stanisław Niemczyk, Przemysław Dyrla

**Affiliations:** 1 Department of Internal Diseases, Nephrology and Dialysis, Military Institute of Medicine, Szaserów str. 128, 04-141 Warsaw, Poland; 2 Department of Endocrinology and Isotope Therapy, Military Institute of Medicine, Szaserów str. 128, 04-141 Warsaw, Poland; 3 Department of Gastroenterology, Military Institute of Medicine, Szaserów str. 128, 04-141 Warsaw, Poland

**Keywords:** Renal perfusion, Doppler, Antihypertensive treatment, Chronic kidney disease, Hypertension

## Abstract

**Purpose:**

Renal cortical perfusion measured in noninvasive, dynamic ultrasonic method is connected with the hemodynamic cardiac properties and renal function. Antihypertensive drugs affect the functioning of the heart and kidneys. The aim of the study was to evaluate the effect of a chronic use of antihypertensive drugs on ultrasound parameters of renal cortical perfusion.

**Methods:**

The study included 56 consecutive patients (49 M + 7 F, age 54.0 ± 13.3) with stable chronic kidney disease and hypertension. Color Doppler dynamic tissue perfusion measurement was used to assess renal cortical perfusion.

**Results:**

Patients were treated with a mean of 2.7 ± 1.4 antihypertensive drugs, of which diuretics accounted for 25%, angiotensin-converting enzyme inhibitors (ACE-I) together with angiotensin receptor blockers (ARB) 24%, beta-blockers (BB) 23%, calcium channel blockers 16%, alpha-1 blockers (α1B) 9% and centrally acting drugs 3%. All investigated groups of drugs correlated significantly with parameters of renal perfusion. In multivariable regression analyses adjusted to age, diuretics were connected with the decrease (*r* = − 0.473) and ACE-I + ARB (*r* = 0.390) with the improvement of proximal and whole renal cortex perfusion (*R*^2^ = 0.28; *p* < 0.001), whereas BB (*r* = − 0.372) and α1B (*r* = − 0.280) independently correlated with worsened perfusion of renal distal cortex (*R*^2^ = 0.21, *p* < 0.01).

**Conclusions:**

The type of antihypertensive therapy had a significant influence on the ultrasound parameters of renal cortical perfusion. Noninvasive, ultrasonic dynamic tissue perfusion measurement method appears to be an adequate tool to assess the impact of drugs on renal cortical perfusion.

## Introduction

Kidneys are one of the best-perfused organs of the body, with the blood flow reaching almost one-fourth of cardiac output. The adequate organ perfusion is prerequisite to maintain normal tissue oxygenation and kidney functions. It is estimated that 85% of the total renal blood flow supplies renal cortex, 14% outer medulla and only 1% inner medulla. Thus, even a slight decrease in perfusion which exceeds renal autoregulation can have a significant impact on the functioning of this organ. Most frequent imaging methods allowing for the quantification of renal perfusion include scintigraphy, and contrast methods such as magnetic resonance imaging (MRI), computed tomography, angiography and ultrasound. However, these examinations are less accessible and burdened with possible complications.

Noninvasive assessment of some aspects of renal hemodynamics is possible in ultrasound examination with the use of Doppler-derived renal resistive index (RI) obtained in the intrarenal segmental arteries [1]. In many studies, RI was widely used in the detection of renal artery stenosis [2], the prediction of revascularization outcomes [3], evaluation of chronic kidney disease (CKD) progression [4], diagnosis of allograft rejection [5], antihypertensive treatment efficacy [6], hypertensive target organ damage [7] and occurrence of iatrogenic arteriovenous fistula [8]. In the recent study, Toledo et al. [9] showed an independent association of high RI with an increased mortality in CKD patients. Although previous studies [10, 11] reported independent relations of RI with age, female sex, body weight, pulse pressure, mean arterial pressure, heart rate, echocardiographic Doppler parameters, renal function and a use of B-blockers, no direct connections of RI with renal vascular resistance and renal perfusion were found. RI seems to be rather a product of a complex interaction between different factors including renal and systemic vascular properties, cardiac hemodynamics and renal parenchymal alterations.

In recent years, it has become possible to assess renal perfusion without the administration of contrast agents using, for example, MRI-ASL (Arterial Spin Labeling) [12] or ultrasound method based on the quantification of the flow visualized by color Doppler, so-called dynamic tissue perfusion measurement (DTPM) [13]. So far, the usefulness of this ultrasonic method in assessing renal perfusion has been demonstrated, among others, in the diagnosis of cardiorenal disturbances [14] or reflux [15] and diabetic nephropathies [16]. The main parameters of perfusion evaluated in this way were significantly associated with both cardiac hemodynamics and kidney function. Antihypertensive drugs influence vascular wall, fluid volume and cardiac function in a direct or indirect manner which result in lowered blood pressure. Therefore, it appears that the use of antihypertensive drugs in the treatment of hypertension should also have an impact on the ultrasound parameters of renal perfusion. The evaluation of the relations between a chronic use of antihypertensive drugs and ultrasound parameters of renal cortical perfusion was the aim of this study.

## Materials and methods

The study included 56 consecutive patients (49 M + 7 F, age 54.02 ± 13.28) with stable chronic kidney disease (CKD) and hypertension treated for at least 30 days, reporting to the Department of Nephrology or Outpatient Clinic, who consented to the study and had a verified antihypertensive drugs regimen. Exclusion criteria included taking nonsteroidal anti-inflammatory drugs, dual or triple renin–angiotensin–aldosterone system blockade, active immunosuppressive therapy, inflammation, hyperkinetic state, uncontrolled hypertension in the interview, atrial fibrillation, acute cardiac and renal diseases, moderate-to-severe heart valve insufficiency or stenosis, heart failure in NYHA III or IV stage, history of renal artery stenosis, CKD stage 5, hydronephrosis, secondary causes of CKD (connective tissue diseases, vasculitis, diabetes mellitus, amyloidosis), systemic cancer and the lack of good-quality ultrasound imaging of renal structures. Despite a negative anamnesis, patients qualified to the study were examined by a physician to exclude signs and symptoms of heart failure (e.g., dyspnea, pulmonary congestion and edemas), significant arrhythmias (mainly atrial fibrillation) and valvular dysfunctions.

### Antihypertensive treatment

Based on the interview collected from each patient, the received antihypertensive drugs were classified into the following groups: (1) angiotensin-converting enzyme inhibitors (ACE-I); (2) angiotensin receptor blockers (ARB); (3) beta-blockers (BB); (4) calcium channel blockers (CCB); (5) diuretics (D); (6) α1-adrenergic receptor blockers (α1B); and (7) centrally acting drugs (CAD). The actual administration of drugs and the length of their application were verified on the basis of ambulatory and hospital documentation of the patients.

### Blood tests and blood pressure monitoring

Serum creatinine (Cr) and cystatin C (Cys) were examined to assess glomerular filtration rate using CKD-EPI (Chronic Kidney Disease Epidemiology Collaboration) formula [17]. To exclude active inflammation, C-reactive protein was estimated. On the day preceding renal perfusion measurement, the 24-h Ambulatory Blood Pressure Monitoring (ABPM-04, Meditech, Hungary) was performed and the mean of 24-h systolic and diastolic blood pressure (SBP, DBP), pulse pressure (PP) and mean arterial pressure (MAP) were recorded.

### Color Doppler sonography

Ultrasound equipment (Logiq P6, GE-Healthcare, Korea) with 4L (2–5 MHz) convex transducer was used to perform renal Doppler ultrasound. All patients underwent the Doppler examination of renal arteries to exclude significant renal artery stenosis. In a few cases, when the imaging of renal arteries was not satisfactory, the renal resistive index (RI) > 0.45 in segmental arteries and the difference between double-sided RI < 0.05 excluded significant narrowing of renal arteries. The renal perfusion was estimated as it was described before [7, 14]. Briefly, we placed a color Doppler frame on the longitudinal section of the mid-segment of right kidney cortex in order to visualize vessels running directly to the transducer. Initially, color Doppler frequency was set on 3.4 MHz and gain was set optimally avoiding artifacts and never changed. In the middle phase of exhalation, the patient was asked to suspend breathing so that the image could be stabilized. Then, 2–3 movie files containing the image sequences in color Doppler, each lasting for at least 3–5 full heart cycles, were recorded in DICOM format. After the examination, the best-quality movie file with recorded flow was transferred to a personal computer, and DTPM was used to quantitatively evaluate perfusion with the use of the PixelFlux (Chameleon-Software, Leipzig, Germany) software package. The region of interest (ROI) was set within the color Doppler frame, in the area between the outer border of medullary pyramids and the kidney surface and contained renal cortex without focal abnormalities. Mean perfusion (MP) intensity (perfusion intensity = flow velocity * flow area/ROI area [cm/s]), which was the mean of arterial perfusion (AP) and venous perfusion (VP) intensities in the total (*t*), proximal (*p*) and distal (*d*) cortical layers, was evaluated [13]. Renal resistive index (RI = (peak systolic − end-diastolic)/peak systolic velocity) in cortical vessels was evaluated separately for arteries and veins in the inner and outer cortexes, as well as in the whole ROI. The ultrasound machine operator did not know the results of the anamnesis and prescribed blood pressure medications.

### Statistics

For statistical analysis, Statistica 12 (StatSoft Inc.) software was used. The administration of ACE-I and ARB was analyzed separately and together as Drugs Acting on Angiotensin (DAA) group. Differences between cortical perfusion parameters were estimated with the use of Wilcoxon’s test. Differences in renal perfusion parameters between the groups were estimated with the use of Mann–Whitney *U* test. Stepwise multivariable linear regression analyses, included all considered antihypertensive medications, were used to determine drugs independently associated with parameters of renal perfusion. Perfusion parameters without a normal distribution were first logarithmized or square-root-transformed, and then, their correlations were evaluated by regression. In order to exclude the effects of age and to find an independent relationship between the administered drugs and perfusion, regression analysis was performed with the adjustment for age.

## Results

Included patients did not present any signs or symptoms of heart failure, significant arrhythmias and valvular dysfunctions. In the examined group of patients, impaired renal function (eGFR 53.0 ± 27.5 ml/min/1.73 m^2^), correct markers of inflammation and controlled blood pressure were observed (Table 1) [18].

**Table 1:** Basic characteristics of study group

Parameter	Mean ± SD (*n* = 56)
Age (year)	54.02 ± 13.28
BMI (kg/m^2^)	28.12 ± 3.65
Cystatin (mg/l)	1.57 ± 0.74
Creatinine (mg/dl)	1.80 ± 0.79
CKD-EPI (Cys-Cr) (ml/min/1.73 m^2^)	53.04 ± 27.48
CRP (mg/dl)	0.64 ± 1.80
SBP (mmHg)	126.82 ± 15.38
DBP (mmHg)	76.98 ± 11.00
MAP (mmHg)	93.67 ± 12.54
PP (mmHg)	49.98 ± 12.54
HR (/min)	67.98 ± 8.84

*BMI* body mass index, *CRP* C-reactive protein, *CKD-EPI* based on cystatin (Cys) and creatinine (Cr) Chronic Kidney Disease Epidemiology formula, *HR* heart rate during ultrasound examination, *MAP, SBP, DBP, PP* data from ABPM: mean arterial pressure, systolic and diastolic blood pressure, pulse pressure

Patients were treated with an a mean of 2.7 ± 1.4 antihypertensive drugs, of which diuretics (*D*) accounted for 25%, drugs acting on angiotensin (DAA; ACE-I and ARB) 24% and beta-blockers (BB) 23% of all of the drugs used (Table 2).

**Table 2:** Analysis of antihypertensive treatment in investigated group

Medication	Patients *n* (%)	Use of antihypertensives (%)
ACE-I	17 (30.4)	11.3
ARB	19 (33.9)	12.7
BB	35 (62.5)	23.3
CCB	24 (42.9)	16.0
Diuretics	37 (66.1)	24.7
A1B	13 (23.2)	8.7
CAD	5 (8.9)	3.3
DAA	36 (64.3)	24.0

*ACE-I* angiotensin-converting enzyme inhibitor, *ARB* angiotensin receptor blocker, *A1B α1* adrenergic receptor blocker (doxazosin), *BB* beta adrenergic receptor blocker, *CCB* calcium channel blocker, *CAD* centrally acting drugs (clonidine, α-methyldopa), *DAA* drugs acting on angiotensin

Six patients were treated with only one drug, whereas two patients received six medications to control their hypertension. The most common 2-drug therapies were combinations of DAA + D and BB + D, which had been previously applied in 33 patients. The most common 3-drug combination was DAA + BB + D having been employed in 14 patients on 3-drug antihypertensive treatment, and another 10 patients on 4–6 drugs regimen. The calculated parameters of perfusion in proximal and distal layers of renal cortex were significantly different (Table 3).

**Table 3:** Results of perfusion measurement of renal cortex

Parameter	Renal cortex (*n* = 57)			*p* value (proximal : distal)
	Total	Proximal	Distal	
MP (cm/s)	0.28 ± 0.21	0.23 ± 0.18	0.05 ± 0.05	< 0.001
AP (cm/s)	0.28 ± 0.23	0.25 ± 0.19	0.04 ± 0.04	< 0.001
VP (cm/s)	0.27 ± 0.26	0.22 ± 0.21	0.06 ± 0.08	< 0.001
ARI (ratio)	0.712 ± 0.130	0.729 ± 0.125	0.887 ± 0.120	< 0.001
VRI (ratio)	0.691 ± 0.164	0.707 ± 0.164	0.815 ± 0.171	< 0.001

*ARI* arterial renal resistive index, *VRI* venous renal resistive index, *MP, AP, VP* mean, arterial, venous perfusion intensity

The analysis of correlation revealed no significant relationships of separately examined ACE-I and ARB with parameters of renal perfusion, except inversely proportional dependence of renal cortical arterial resistive index (ARI) with ACE-I (*r* = − 0.283; *p* < 0.05). More correlations with the parameters of renal perfusion were observed for combined ACE-I and ARB (Table 4).

**Table 4:** Coefficients of significant correlations between renal cortical perfusion parameters and antihypertensive drugs

Parameter	DAA	BB	CCB	D	CAD	α1B	Age
MP (cm/s)		− 0.393	− 0.347	− 0.383			− 0.346
AP (cm/s)		− 0.392	− 0.286	− 0.368			− 0.325
VP (cm/s)	0.358	− 0.279	− 0.342	− 0.314	− 0.364		− 0.273
p-MP(cm/s)		− 0.381	− 0.381	− 0.375			− 0.377
p-AP (cm/s)		− 0.365	− 0.297	− 0.353			− 0.347
p-VP (cm/s)	0.373	− 0.272	− 0.374	− 0.308	− 0.333		− 0.293
d-MP (cm/s)		− 0.371		− 0.314			− 0.347
d-AP (cm/s)		− 0.347	− 0.294			− 0.285	− 0.305
d-VP (cm/s)		− 0.276		− 0.305	− 0.283		
ARI		0.332					0.506
p-ARI		0.325					0.475
d-ARI							0.454
VRI	− 0.283						0.283
p-VRI	− 0.300						0.324
d-VRI	− 0.354	0.325					0.290

*ACE-I* angiotensin-converting enzyme inhibitor, *(p-/d-) ARI* (proximal/distal) arterial renal resistive index, *ARB* angiotensin receptor blocker, *α1B* α1 adrenergic receptor blocker (doxazosin), *BB* β-blocker, *CCB* calcium channel blocker, *CAD* centrally acting drug (clonidine, α-methyldopa), *D* diuretics, *DAA* drug acting on angiotensin (ACE-I + ARB), *MP, AP, VP* mean, arterial, venous perfusion intensity, *(p-/d-) MP, AP, VP* (proximal/distal) mean, arterial, venous perfusion intensity, *(p-/d-) VRI* (proximal/distal) venous renal resistive index, *empty box* correlation not significant

With the exception of DAA, the majority of antihypertensive drugs were negatively related to renal perfusion, and the use of BB additionally correlated with higher resistive index (RI) values. However, a similar relationship with markers of renal perfusion was also found for age. Only venous perfusion (VP) of proximal renal cortex was significantly connected with heart rate during the examination (*r* = 0.29; *p* = 0.039). Moreover, investigated renal perfusion parameters were not correlated with sex, BMI and actual heart rate. After adjusting for age, regression analysis showed an independent influence of antihypertensive drugs on renal cortical perfusion parameters achieved in DTPM (Table 5).

**Table 5:** Results of multivariable regression: significant regression coefficients and estimated independent influence of antihypertensive drugs on renal perfusion parameters

Parameter	ACE-I	ARB	DAA	BB	CCB	D	α1B	R^2^	*p* value
MP (cm/s)	0.426**	0.339*	x			− 0.473***		0.29	< 0.001
	x	x	0.390**			− 0.471***		0.28	< 0.001
AP (cm/s)						− 0.369**	− 0.295*	0.23	< 0.001
VP (cm/s)	0.496***	0.401**	x			− 0.446***		0.31	< 0.001
	x	x	0.448***			− 0.444***		0.30	< 0.001
p-MP (cm/s)	0.394**	0.343*	x			− 0.462***		0.27	< 0.001
	x	x	0.377**			− 0.461***		0.27	< 0.001
p-AP (cm/s)					− 0.277*	− 0.308*		0.21	< 0.01
p-VP (cm/s)	0.492***	0.446**	x			− 0.418***		0.31	< 0.001
	x	x	0.480***			− 0.416***		0.29	< 0.001
d-MP (cm/s)				− 0.372**			− 0.280*	0.21	< 0.01
d-AP (cm/s)				− 0.319*			− 0.376**	0.24	< 0.001
d-VP (cm/s)	0.374*	0.308*	x			− 0.427**		0.23	< 0.01
	x	x	0.347**			− 0.425**		0.23	< 0.01
ARI	− 0.270*	x	x	0.335**				0.18	< 0.01
	x			0.327*				0.11	< 0.05
p-ARI				0.330*				0.11	< 0.05
d-ARI									
VRI	− 0.276*		x					0.08	< 0.05
	x	x	− 0.331*					0.11	< 0.05
p-VRI	− 0.309*		x					0.09	< 0.05
	x	x	− 0.324*					0.10	< 0.05
d-VRI			x	0.271*				0.07	< 0.05
	x	x	− 0.323*	0.283*				0.18	< 0.01

*ACE-I* angiotensin-converting enzyme inhibitor, *(p-/d-) ARI* (proximal/distal) arterial renal resistive index, *ARB* angiotensin receptor blocker, *α1B* α1 adrenergic receptor blocker (doxazosin), *BB* β-blocker, *CCB* calcium channel blocker, *D* diuretics, *DAA* drugs acting on angiotensin (ACE-I + ARB), *(p-/d-) MP, AP, VP* (proximal/distal) mean, arterial, venous perfusion intensity, *(p-/d-) VRI* (proximal/distal) venous renal resistive index, *x* variable not included to the regression analysis and *empty box* connection not significant

Significance of regression coefficients: *p < 0.05, **p < 0.01, ***p < 0.001

The use of DAA was independently associated with an improved mean cortical perfusion (MP), probably due to lowered arterial resistance (expressed as ARI) and increased venous perfusion. The application of diuretics (> 56% of loop diuretics) correlated with a decrease in arterial and venous perfusion particularly expressed in the proximal cortical layer, without changing the perfusion resistance. When we compared patients treated with DAA but not with diuretics with those treated with diuretics but not with DAA, mean perfusion intensity was significantly lower in the diuretic group (0.277 ± 0.194 vs 0.116 ± 0.058, *p* < 0.05). The total influence of drugs acting on angiotensin and diuretics on MP was estimated at 28%, with the statistical power of 0.95 for the probability of type I error = 0.05. Necessary sample size to achieve significance (*p* < 0.05) was 34 patients for the statistical power of the test of 0.8, and 43 patients for the power of 0.9. In a subgroup analysis, the mean cortical perfusion was significantly lower in patients treated with loop diuretics than with thiazide or thiazide-like ones (MP 0.36 ± 0.21 vs 0.15 ± 0.11 cm/s, *p* < 0.001). However, these groups differed with the renal function (CKD-EPI (Cys-Cr) 68.2 ± 24.6 vs 33.5 ± 17.1 ml/min/1.73 m^2^; *p* < 0.001), but not with the age. After adjusting to CKD-EPI(Cys-Cr), univariable linear regression analysis showed significant correlation between the use of loop diuretics and diminished mean cortical perfusion (*r* = − 0.46, *R*^2^ = 0.21; *p* < 0.001).

Investigated patients were treated with different beta-blockers (71% with betaxolol, bisoprolol and metoprolol and 29% with carvedilol and nebivolol). The use of BB was associated with an increased proximal and total arterial resistive index (p-ARI, ARI) and a reduction in distal mean and arterial perfusion (d-MP, d-AP). There were no significant differences in all investigated renal perfusion parameters between groups of β_1_-selective (betaxolol, bisoprolol and metoprolol) and vasodilatory BB (carvedilol and nebivolol).

As vasodilatory drugs, calcium channel blockers and alpha-1 receptor blockers (α1B) were associated with a reduction in perfusion in renal cortical arteries. Based on multivariable regression analysis, no significant impact of centrally acting agents (clonidine, alpha-methyldopa) on Doppler parameters of renal perfusion was found.

## Discussion

In the present study, we show for the first time a significant influence of particular groups of antihypertensive drugs on renal perfusion assessed by DTPM. In previous studies [7, 14], we have shown a significant independent association of ultrasound parameters of renal cortical perfusion with the renal function, mean arterial pressure and cardiac systolic function. In these works, we lacked an analysis of the influence of antihypertensive drugs or compared two groups, which did not differ in the method of antihypertensive treatment, renal function and blood pressure, so the impact of the drugs could be omitted. In the present study, we demonstrated a significant independent association of renal perfusion parameters assessed by DTPM with the type of antihypertensive drugs. The correlations demonstrated in our work correspond with current knowledge based on invasive methods [19]. The drugs blocking the activity of angiotensin II (ACE-I, ARB) preferentially dilate the efferent arteriole in renal glomerulus and thus decrease renal vascular resistance (RVR), reduce intraglomerular capillary pressure and increase renal blood flow. ACE-I are the primary antihypertensive drugs with the most nephroprotective properties. Degaute et al. [20] showed a significant decrease in RVR in patients chronically treated with lisinopril. Moreover, based on the example of 20 patients treated for eight weeks with enalapril, De Cesaris et al. [21] found a significant increase in effective renal plasma flow and a decrease in RVR. In the study by Ritt et al. [22], a two-week therapy with telmisartan resulted in the significant increase in renal perfusion measured in MRI-ASL. According to these observations, in our study the use of ACE-I and ARB was independently associated with the significant increase in renal cortical perfusion in DTPM. In addition, the ACE-I treatment was associated with lower values of arterial resistive index.

Beta-blockers are another major group of antihypertensive drugs frequently used in concomitant cardiac diseases, although the mechanism of antihypertensive effect of these agents is not precisely defined. Blockade of β_1_-receptors in renal juxtaglomerular cells can inhibit renin release and decrease angiotensin production. By the attenuation of sympathetic nerves stimulation mediated by catecholamines, BB decrease cardiac output and blood pressure and can decrease eGFR and renal blood flow. Although it is believed that, unlike non-selective BB, β_1_-selective blockers do not increase renal vascular resistance and reduce RBF, in the study by study Koch et al. [23], intake of metoprolol was associated with a decrease in RBF of approx. 11% and an increase in RVR of approx. 10%. Similar conclusions were drawn by Schmieder et al. [24], who observed in 43 patients that the use of metoprolol was related to an increased peripheral resistance and a reduction in renal plasma flow. The authors suggested that the imbalance between blocked β_1_-receptors and active vasoconstrictive α-receptors in peripheral arteries is responsible for the increase in vascular resistance. In accordance with the above-mentioned results remain our observations, in which the use of BB (mainly metoprolol and bisoprolol) was connected with an increased RI and a decrease in renal cortical perfusion, particularly expressed in the distal cortex. Although the nature of cortical RI measured in arcuate and interlobular arteries can differ from ‘segmental’ RI, the use of BB in large population studies was an independent factor for the increase in RI in segmental arteries [9–11]. In the study by Leeman et al. [25], metoprolol reduced RBF and was significantly worse in reducing RVR than the carvedilol. In the experimental study [26], the use of the vasodilatory BB nebivolol increased eGFR and renal plasma flow. Nevertheless, probably due to the small group of patients treated with vasodilatory BB (*n* = 10), we did not find any significant differences between β_1_-selective and vasodilatory BB in investigated renal perfusion parameters.

In the presented study, the administration of diuretics in patients with hypertension and CKD was related to a reduced renal cortical perfusion. However, it was not associated with a change in RI. The chronic use of thiazide, thiazide-like and loop diuretics results in extracellular fluid volume depletion and increase in renin release, both more pronounced in loop diuretics. In addition, loop diuretics attenuate renal autoregulation by tubuloglomerular feedback blockade, dilate glomerular afferent arteriole and dilate veins by stimulating of prostaglandin production. For this reason, the result of a chronic use of most diuretics on renal hemodynamics is similar to the activity of vasodilators [19]. In the study by Hung et al. [27], patients with CKD and volume overload significantly more frequently than those without overhydration, used the indapamide (thiazide-like drug), which unfortunately causes a decrease in renal perfusion. In our study, chronic treatment with loop diuretics was associated with the decrease in renal perfusion, independent of renal function. However, except for eGFR, we did not study the influence of other factors modifying renal perfusion as well as indications for loop diuretics prescription. Nevertheless, Wang et al. [28] performing MRI-ASL before and after the intravenous dose of furosemide found the significant decrease in the cortical and medullar renal blood flow after the treatment.

It is clear that our results of the relationship between ultrasound renal cortical perfusion assessment and chronically used antihypertensive drugs are consistent with current knowledge. Another advantage of the method is a separate evaluation of venous and arterial perfusion, and the ability to analyze perfusion of different layers in renal cortex. Perfusion parameters of proximal and distal cortex have differed significantly, so it is reasonable to consider the influence of antihypertensive drugs on these parameters separately. Recently, ASL-MRI has become a more and more frequently used noninvasive and more objective method assessing renal perfusion, also in connection with the drugs [28]. However, unlike DTPM, this method cannot differentiate between arterial and venous perfusion.

Despite the promising results, our work has some limitations resulting mainly from the study protocol. In this work, we did not intend to check the reproducibility of DTPM. Based on the similarity of used ultrasound methods and further software-dependent processing, we could expect, approximated to RI evaluation, values of intraobserver (2.07–5.1%) and interobserver (3.61–6.2%) variability [29]. We adjusted DTPM results mainly for age. Previous studies reported an independent association of RI with demographic and hemodynamic factors as well as with renal function [10, 11]. However, RI expresses rather renal vascular impedance than perfusion [30]. Moreover, due to increasing downstream resistance and different perivascular tissue properties, RI achieved in segmental arteries should differ from cortical RI measured in arcuate and interlobular intrarenal arteries [31]. These differences are likely to affect some associations and in the presented study cortical RI correlated with age, but not with heart rate and BMI. Another topic which should be mentioned arises from different equations describing RI and the intensity of renal perfusion, despite similar examination methods [13]. Thus, the results of previous studies concerning RI associations cannot be easily referred to DTMP parameters in renal parenchyma. Moreover, it is possible that the prevalence of male sex influenced the correlations of perfusion parameters, so that the results cannot be generalized. With an exception of the probable impact of renal function on the choice of prescription of thiazide/thiazide-like or loop diuretics, we did not analyze differences between individual indications for different classes of antihypertensive drugs.

Another aspect is a treatment of hypertension with more than one drug, which brings a possibility of mutual amplification or attenuation of drug influence on renal vasculature. Although these influences were taken into consideration account in the multivariable regression analysis, our results may not contain complete information on the relationship of the individual drugs with renal cortical perfusion assessed by ultrasound. This issue, however, can be a reason for further research.

## Conclusions

The type of antihypertensive therapy has a significant influence on the ultrasound parameters of renal cortical perfusion. Noninvasive, ultrasonic dynamic tissue perfusion measurement method appears to be an adequate tool to assess the impact of drugs on renal cortical perfusion.
